# Chromium(III), chromium(VI) and cobalt release from leathers produced in Nicaragua

**DOI:** 10.1111/cod.13165

**Published:** 2018-11-28

**Authors:** Yolanda S. Hedberg, Zheng Wei, Federico Moncada Chévez

**Affiliations:** ^1^ Division of Surface and Corrosion Science, Department of Chemistry, School of Engineering Sciences in Chemistry, Biotechnology, and Health KTH Royal Institute of Technology Stockholm Sweden; ^2^ Public Health Department, Faculty of Medical Science National Autonomous University of Honduras Tegucigalpa Honduras; ^3^ Central American Network of Information and Advice Centres in Toxicology (RedCIATOX) Tegucigalpa Honduras; ^4^ Centre for Research and Development in Health, Labour and Environment (CIDSTA) Tegucigalpa Honduras

**Keywords:** allergic contact dermatitis, chromium, chromium(VI), cobalt, exposure analysis, leather

## Abstract

**Background:**

Leather exposure has been associated with chromium (Cr) and cobalt (Co) contact dermatitis. Cr(VI) in leather is now restricted to <3 mg/kg in the EU. Cr(III) is not restricted.

**Objectives:**

To analyse 29 differently coloured Cr‐tanned leather samples from two Nicaraguan tanneries, and to compare their release of Cr, Cr(VI) and Co with that of leathers produced in Europe.

**Methods:**

Cr, Cr(VI) and Co were extracted in phosphate buffer for 3 hours at 25°C according to EN ISO 17075. Atomic absorption spectroscopy and spectrophotometry were used for detection of the metals in phosphate buffer.

**Results:**

There was no difference in total Cr or Cr(VI) release between European and Nicaraguan leathers. There was no association between Cr(VI) and total Cr release. Co was released primarily from leathers of one tannery. Cr(III) was released in significantly higher amounts than Cr(VI).

**Conclusions:**

Future investigations and regulations should focus on Cr(III) and Co as well as on Cr(VI).

## 1 INTRODUCTION

Recent, mostly European, studies have suggested a strong association between leather exposure and contact dermatitis caused by chromium (Cr).^1^, ^2^, ^3^, ^4^, ^5^, ^6^ Most leather is Cr‐tanned worldwide, but several alternatives exist.^7^, ^8^ Since 2015, hexavalent Cr (Cr[VI]) has been restricted to <3 mg/kg in leather in the EU by the REACH legislation, according to an extraction test in phosphate buffer for 3 hours.^9^ Low‐quality chemicals, fish oils and low‐quality tannery methods have been suggested by the tannery industry and some studies to cause the formation of Cr(VI) in Cr‐tanned leather.^8^, ^10^ The presence of reducing agents and conditions during usage and storage also determine the release of Cr(VI) from Cr‐tanned leather.^8^, ^11^, ^12^, ^13^

Currently, the majority of leather is produced in developing countries, while leather production in developed countries is decreasing.^14^ Historically, the tanning industry was characterized by small or medium family businesses.^15^ An increasing volume of production is now sought at a lower cost, which means that the number of informal contracts is increasing. León in Nicaragua is no exception, as there is high labour demand, with little or no skilled labour, in the field of leather production. The value chain of leather in Nicaragua includes cattle ranches, slaughterhouses, collectors, tanneries (which are concentrated in Granada, Chinandega, León, and Managua), factories and craft workshops producing footwear, leather goods, and saddlery, and fur exporters. Of all skins in the country, 24% are exported salted, 38% are exported semi‐tanned to chrome or blue (Wet Blue), and another 38% are processed in the tanneries to produce finished tanned leather for use in the footwear, leather goods and saddlery industry.^16^ In 2014, in the first 9 months of the year, footwear worth 15.9 million $US was exported, 26% more than in all of 2013.^17^ In 2015, the footwear industry reached the record figure of 9 million pairs of manufactured shoes, with revenues of >50 million $US.^18^ Hence, the leather industry is of utmost economic importance to Nicaragua.

The objectives of this study were to analyse a number of Nicaraguan leather samples obtained from two different tanneries in terms of their Cr, Cr(VI) and cobalt (Co) release, and to compare the amounts released with those released by some European leathers.

## 2 MATERIALS AND METHODS

### 2.1 Collection sites

The collection was conducted in the municipality of León, which is located in the western part of Nicaragua. León is located at latitude 12°26″ north and longitude 86°53″ west. Forty per cent of registered tanning companies nationwide are located in this municipality.^16^

According to data obtained from the Ministry of Family, Community, Cooperative and Associative Economy (MEFCCA), in the municipality of León there are 32 registered companies in which tannery work is carried out; however, only seven of them perform the entire process, and the remaining 25 are responsible for providing services, carrying out some parts of the process that involves the production of leather, but without involving exposure to chemical agents and to chromium.

The seven companies that carry out the entire process are medium and small in relation to the number of workers, and all are of a family nature. In each of them, ∼10 workers work in a stable way, mostly informal, by verbal agreement, with seniorities of up to 10 years. The hiring of other workers is very variable in terms of number, as it depends on the workload at the time and the skill levels of the worker in the different job tasks as perceived by the tannery owner, which is not formally verified.

Among all of the tanneries available, three owners (two tanneries) agreed to have their finished leather tested. All owners were concerned about the health of their workers, as social security is non‐existent for informal workers, and costs are often paid by the owners themselves. Only one of the tannery owners had a degree in agronomics, with some knowledge of the whole process and better knowledge of new strategies in the tannery industry (eg, vegetable tanning and water recycling). The Nicaraguan government does not currently regulate the use of chemicals directly in this industry. Instead, the MEFCCA allows local families engaged in this type of work to easily import chemicals with practically no regulation at all.

No remuneration was given or asked for. A disclosure agreement was signed by the researchers and the owners. There was no conflict of interest among the researchers, tannery owners, or government.

### 2.2 Collection of leather samples

**Figure 1:**
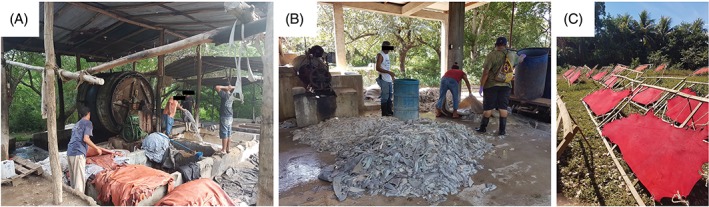
Workplace images from the Nicaraguan tanneries (**A, B**) and finished (coloured) leather drying in the sun (**C**)

The leather pieces obtained from each of the tannery owners were kept either in their houses without any cover or in inadequate storage spaces in the workplace. None of these leathers was kept isolated by the producers, and were stored mostly in bulk according to colour or the final product to be made (Figure 1A). Leather pieces of at least 4 × 4 cm were requested. Crushed Wet Blue (mainly Cr‐tanned) leather was also collected. Each sample was labelled according to its colour or purpose, placed in a sealed plastic bag, and then sent for laboratory analysis to KTH, Sweden.

### 2.3 Leather sample preparation

All leathers (Table 1) were stored for 1 week in a desiccator (<10% relative humidity) at room temperature prior to testing for Cr release. This has previously been shown to be equivalent to conditions of different temperatures and a relative humidity of <35%, which enables the release of Cr(VI), if present.^6^, ^13^ Independent duplicate samples of each leather material produced in Nicaragua were tested, in addition to a reference material, which has been extensively tested before and was produced in Europe.^6^ For comparison, other leathers produced in Europe and previously tested under similar conditions were also included in the data analysis.^11^, ^12^, ^13^, ^19^

**Table 1:** Origin, sample ID and colour of leather samples, as well as weight and surface area during extraction in phosphate buffer

Origin	Sample ID	Colour	Weight (g)	Surface area (cm^2^)	Volume of phosphate buffer (mL)
Tannery 1	A	Yellow	0.18‐0.19	5.7	10
	B	Green	0.26‐0.28	5.7	10
	C	Grey	0.17	5.1	10
	D	Blue	0.18‐0.19	5.7	10
	E	Purple	0.22	5.7	10
	F	Brown	0.20‐0.23	5.7	10
	G	Brown	0.11	5.7	10
	H	Red	0.27	5.7	10
	I	Black	0.24	5.7	10
	J	Black	0.40‐0.44	5.2	10
	K	Grey	0.20	Shredded	10
Tannery 2	L	Brown	0.27‐0.31	5.7	10
	M	Green	0.24‐0.33	5.7	10
	N	Brown	0.21‐0.24	5.7	10
	O	Pink	0.17‐0.19	5.7	10
	P	Green	0.33	5.7	10
	Q	Purple	0.16	5.7	10
	R	Brown	0.20‐0.24	5.7	10
	S	Orange	0.22	5.7	10
	T	Black	0.23‐0.27	5.7	10
	U	Brown	0.21‐0.25	5.7	10
	V	Red	0.24‐0.26	5.7	10
	W	Black	0.26‐0.28	5.7	10
	X	Brown	0.27‐0.28	5.7	10
	Y	Orange	0.24‐0.28	5.7	10
	Z	Brown	0.14	5.7	10
	AA	Black	0.14‐0.16	5.7	10
	BB	Black	0.35‐0.36	5.7	10
	CC	Black	0.30‐0.35	5.7	10
Europe	Ref.	Grey	0.15‐0.16	5.7	10
	Cr^Cr^	Brown	0.99‐1.4	22‐28	50
	Cr^veg^	Brown	0.96‐1.1	16‐18	50
	Veg^veg^	Orange	21‐23	7.6	50

Abbreviations: Ref., Cr‐tanned leather; Cr^Cr^, Cr‐tanned and coloured leather; Cr^veg^, Cr‐ and vegetable‐tanned coloured leather; Veg^veg^, vegetable‐tanned reference leather.

Ultrapure water (resistivity of 18.2 MΩ cm; Millipore, Solna, Sweden) was used as the solvent for all solutions, and all equipment was acid‐cleaned (10% HNO_3_ for at least 24 hours) prior to use, and then rinsed four times with ultrapure water. All leather samples and two blank samples (without any leather) were extracted in 22.8 g/L K_2_HPO_4_.3H_2_O phosphate buffer of pH 8.1 for 3 hours at 25°C in darkness, according to ISO 17075,^9^ in a bilinearly shaken incubator (22 cycles/minute, 12° inclination). After extraction, the solution was frozen prior to Cr(VI) analysis or acidified (pH < 2) prior to total Cr analysis with atomic absorption spectroscopy (AAS). The freezing procedure, which is not specified in ISO 17075,^9^ is necessary if the samples are not to be analysed immediately, in order to prevent changes in Cr speciation in the sample during storage.

### 2.4 Atomic absorption spectroscopy

The total amount of Cr released was determined by the use of AAS (AAnalyst 800; Perkin Elmer, Upplands Väsby, Sweden) with calibration standards of concentrations 0, 5, 15, and 45 mg/L Cr (in 1% HNO_3_). The limit of detection (LOD) was estimated to be 0.012 mg Cr/L. All sample solution concentrations in this study were significantly higher than the LOD, and the blank concentrations were lower than the LOD. After four samples had been measured, quality controls of known concentrations were measured. If the measured control sample deviated by >10%, recalibration was performed. Similarly, the total amount of Co released was also determined by the use of AAS in the flame mode, with a detection limit of 0.015 mg/L Co.

### 2.5 Spectrophotometry

The amount of Cr(VI) in the extractant was determined with spectrophotometry (Jenway 6300, Staffordshire, UK), utilizing the pink colour of the complex of Cr(VI) and diphenylcarbazide (DPC),^20^ with an absorption maximum at 540 nm. As in our previous studies^6^, ^11^, ^12^, ^13^, ^19^ and in accordance with ISO 17075,^9^ the solutions were mixed in the ratio of 96 vol% sample, 2 vol% phosphoric acid, and 2 vol% DPC solution. The concentration of phosphoric acid was 70 vol%, in water and the DPC solution was composed of 1.0 g of 1,5‐DPC in 100 mL of acetone, acidified with one drop of glacial acetic acid. It was freshly prepared and colourless (non‐oxidized). The calibration was based on 0, 125, 247.5, 495 and 990 μg of Cr(VI)/L in phosphate buffer. The LOD was considered to be 30 μg of Cr(VI)/L (highest blank concentration plus three times the highest SD of all blanks). Calibration curves were linear (*R* ^2^ = 0.9959).

ISO 17075^9^ specifies a solid‐phase extraction procedure to remove the dyes from the extraction fluid, but this was not applied in this study, owing to the risk of simultaneous Cr(VI) removal. We investigated membrane filtration with pores with sizes down to 20 nm, but could not remove the colours of coloured solution samples, and so did not apply any filtration, owing to the risk of removing Cr(VI) from solution. Solution samples that were coloured were also investigated by standard addition, that is, adding known amounts of Cr(VI) to the solution sample. Similarly to what was found in a previous study,^19^ the standard addition resulted in fluctuating results because of interference and, possibly, reactions with DPC and dyes.

### 2.6 Statistical analysis

To determine whether differences in the amounts of released metals between different groups were statistically significant, the Student’s *t* test, for unequal variance and unpaired data, was applied by the use of kaleidagraph v. 4.0 software.

## 3 RESULTS

**Figure 2:**
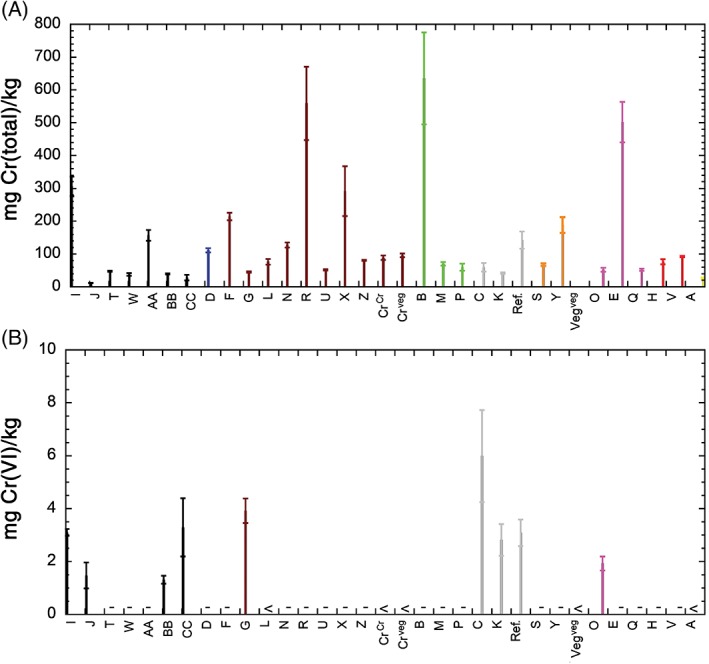
Total released Cr (**A**) and released Cr(VI) (**B**) in mg per dry mass of leather (kg) after extraction for 3 hours in phosphate buffer (pH 8.0) at 25°C. Note that 3 mg/kg Cr(VI) is the restriction limit in REACH for leather. The data are ordered according to the colour of the leather, whereby all leathers were coloured except for the grey leathers (natural colour for Cr‐tanned leather) and the vegetable‐tanned reference leather (Veg^veg^) (not Cr‐tanned). “<” indicates that the value was below the limit of detection. “‐” indicates that the value was not measurable, owing to colour interference. Ref., Cr‐tanned leather; Cr^Cr^, Cr‐tanned coloured leather; Cr^veg^, Cr‐ and vegetable‐tanned coloured leather, Veg^veg^, vegetable‐tanned reference leather

**Figure 3:**
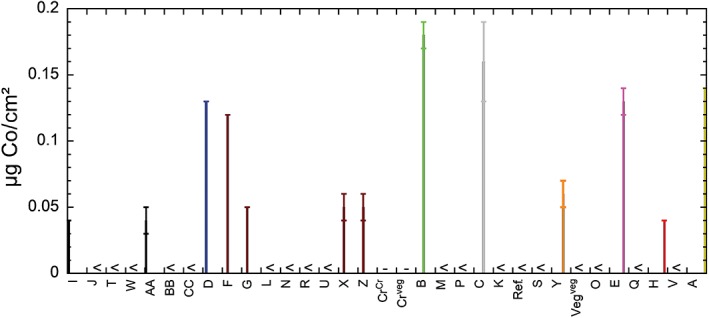
Released Co in μg per cm^2^ of leather after extraction for 3 hours in phosphate buffer (pH 8.0) at 25°C. The data are ordered according to the colour of the leather, whereby all leathers were coloured except for the grey leathers (natural colour for Cr‐tanned leather) and the vegetable‐tanned reference leather (Veg^veg^) (not Cr‐tanned). “<” indicates that the value was below the limit of detection. “‐” indicates that the value was not measured. Ref., Cr‐tanned leather; Cr^Cr^, Cr‐tanned coloured leather; Cr^veg^, Cr‐ and vegetable‐tanned coloured leather, Veg^veg^, vegetable‐tanned reference leather

Table 2 shows the total amounts of Cr, Cr(VI) and Co released from the leather samples during 3 hours of extraction in phosphate buffer at 25°C, normalized to either the surface area or the dry mass of the leather. Normalization to the surface area is of higher relevance to skin contact and for comparing different leather samples, as it is the surface of the leather that is most important for the release of Cr.^11^ Normalization to the dry mass of the leather is relevant from a regulatory perspective. The vegetable‐tanned reference leather, which is included for comparison, did not release any detectable amounts of total Cr, Cr(VI), or Co. All Cr‐tanned leathers released significantly higher amounts of total Cr (both Cr[III] and Cr[VI]) than the detection limit of this study. The total amount of Cr released was 0.89 to 30.4 μg/cm^2^ and 12 to 635 mg/kg (Figure 2A). No statistically significant differences were found between the amounts of total Cr released from leathers of tannery 1 and of tannery 2 (*P* = 0.34), or between Nicaraguan and European leathers (*P* = 0.36). The release of Cr(VI) was not measurable for a number of coloured leather samples, owing to interference of the colour dyes with our method. It was also below the detection limit for four other Cr‐tanned leathers. All three grey leathers (Nicaraguan and European), which are not coloured, released Cr(VI) at amounts between 2.8 and 6.0 mg/kg, which are close to, or higher than, the restriction limit of 3 mg/kg (Figure 2B). Furthermore, four of six black leathers released Cr(VI) at amounts between 1.3 and 3.3 mg/kg. The other two black leathers interfered with our test, and were not measurable. One brown (3.9 mg/kg) and one purple (1.9 mg/kg) leather also released detectable amounts of Cr(VI), whereas most other brown, green, purple, red and orange leathers were not measurable, owing to colour interference. The measurable leathers that released non‐detectable amounts of Cr(VI) were all brown (five leathers, both from Nicaragua and Europe) or yellow (one Nicaraguan leather). The amount of released Co was similar to or lower than the amount of released Cr(VI). There was no clear influence of the colour of the leather (Figure 3). There was, however, a clear difference in Co release between leathers from tannery 1 and tannery 2 (*P* = 0.002).

**Table 2:** Released total Cr and Cr(VI) normalized to the surface areas of the exposed leather samples or their dry masses, respectively; mean values ± SDs of at least duplicate samples are given

Tannery	Sample ID	Total Cr (μg/cm^2^)	Total Cr (mg/kg)	Cr(VI) (μg/cm^2^)	Cr(VI) (mg/kg)	Co (μg/cm^2^)	Co (mg/kg)
Tannery 1	A	0.89 ± 0.080	27 ± 1.5	<LOD		0.14 ± 0	4.3 ± 0.07
	B	30 ± 8.6	635 ± 140	NM		0.18 ± 0.01	3.7 ± 0.4
	C	2.0 ± 0.47	60 ± 13	0.20 ± 0.060	6.0 ± 1.7	0.16 ± 0.03	4.7 ± 0.9
	D	3.6 ± 0.46	111 ± 6.6	NM		0.13 ± 0	4.1 ± 0.3
	E	20 ± 2.7	502 ± 62	0.080 ± 0.010	1.9 ± 0.26	0.13 ± 0.01	3.3 ± 0.2
	F	7.9 ± 0.34	215 ± 11	NM		0.12 ± 0	3.1 ± 0.4
	G	0.89 ± 0.040	45 ± 1.8	0.08 ± 0.010	3.9 ± 0.46	0.05 ± 0	2.5 ± 0.02
	H	3.6 ± 0.37	77 ± 7.6	NM		0.04 ± 0	0.92 ± 0.02
	I	13 ± 1.2	306 ± 30	0.13 ± 0	3.1 ± 0.13	0.04 ± 0	0.84 ± 0.06
	J	0.96 ± 0.010	12 ± 0.74	0.12 ± 0.050	1.5 ± 0.49	<LOD	<LOD
	K	NM	42 ± 2.7	NM	2.8 ± 0.60	<LOD	<LOD
Tannery 2	L	3.9 ± 0.15	76.4 ± 8.8	<LOD	<LOD	<LOD	<LOD
	M	3.5 ± 0.45	70 ± 5.3	NM	NM	<LOD	<LOD
	N	5.1 ± 0.77	127 ± 7.9	NM	NM	<LOD	<LOD
	O	1.6 ± 0.030	52 ± 6.2	NM	NM	<LOD	<LOD
	P	3.4 ± 0.61	60 ± 11	NM	NM	<LOD	<LOD
	Q	1.5 ± 0.050	52 ± 3.4	NM	NM	<LOD	<LOD
	R	21 ± 1.4	559 ± 112	NM	NM	<LOD	<LOD
	S	2.6 ± 0.090	68 ± 3.5	NM	NM	<LOD	<LOD
	T	2.1 ± 0.26	48 ± 1.6	NM	NM	<LOD	<LOD
	U	2.1 ± 0.34	52 ± 2.0	NM	NM	<LOD	<LOD
	V	4.0 ± 0.24	92 ± 1.8	NM	NM	<LOD	<LOD
	W	1.8 ± 0.27	38 ± 3.9	NM	NM	<LOD	<LOD
	X	14 ± 3.8	292 ± 76	NM	NM	0.05 ± 0.01	0.95 ± 0.2
	Y	8.7 ± 2.0	188 ± 24	NM	NM	0.06 ± 0.01	1.4 ± 0.3
	Z	2.0 ± 0.030	81 ± 1.5	NM	NM	0.05 ± 0.01	2.0 ± 0.6
	AA	4.0 ± 0	157 ± 16	NM	NM	0.04 ± 0.01	1.4 ± 0.4
	BB	2.5 ± 0.16	39 ± 1.6	0.080 ± 0.010	1.3 ± 0.15	<LOD	<LOD
	CC	1.6 ± 0.30	28 ± 8.7	0.19 ± 0.080	3.3 ± 1.1	<LOD	<LOD
Europe	Ref.	3.9 ± 0.52	143 ± 26	0.080 ± 0.020	3.1 ± 0.50	<LOD	<LOD
	Cr^Cr^	4.3 ± 0.20	89 ± 7.0	<LOD	<LOD	N/M	N/M
	Cr^veg^	5.8 ± 0.38	96 ± 5.7	<LOD	<LOD	N/M	N/M
	Veg^veg^	<LOD	<LOD	N/M	N/M	<LOD	<LOD

Abbreviations: Ref., Cr‐tanned leather; Cr^Cr^, Cr‐tanned coloured leather; Cr^veg^, Cr‐ and vegetable‐tanned coloured leather; NM, not measurable; <LOD, lower than the limit of detection; Veg^veg^, vegetable‐tanned reference leather.

**Figure 4:**
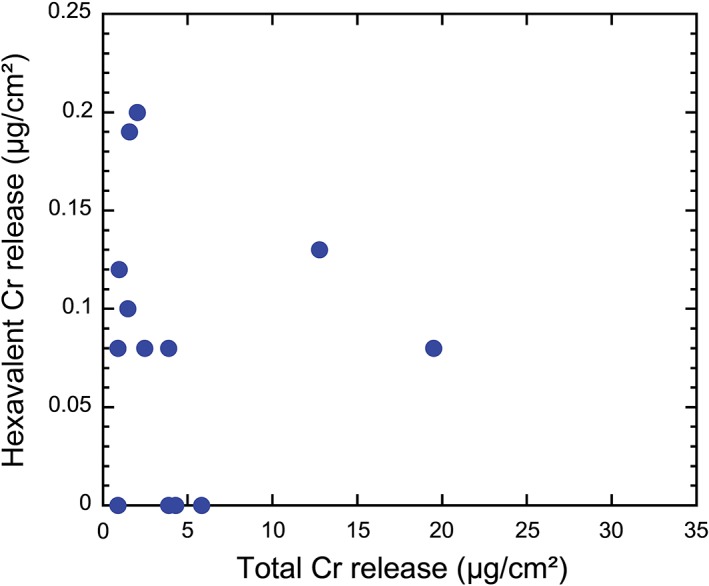
Released Cr(VI) vs released total Cr in μg per cm^2^ of leather after extraction for 3 hours in phosphate buffer (pH 8.0) at 25°C. Cr(VI) values lower than the limit of detection are shown as zero values in this graph. Results from samples for which Cr(VI) values were not measurable because of colour interference are excluded

There was no association between Cr(VI) relase and total Cr release (Figure 4).

## 4 DISCUSSION

The European leathers investigated in this study have been characterized in several studies previously,^6^, ^11^, ^13^, ^19^ and were also included in a recently published use‐test study.^6^ In agreement with those studies, all grey (non‐coloured) Cr‐tanned leathers in this study released detectable amounts of Cr(VI) that were higher than the restriction limit of 3 mg/kg. Furthermore, most, but not all, of the brown leathers (independently of origin) did not release any detectable amounts of Cr(VI). This has previously been explained by the reducing capacity of other chemicals released from that leather.^19^ There was, otherwise, little influence of the leather colours on their release of either total Cr, Cr(VI), or Co. Some colours interfered with our method and prevented the measurement of Cr(VI). It is possible that a solid‐phase extraction, as specified in ISO 17075, would have solved this experimental problem, although the risk of simultaneous Cr(VI) removal would probably not have been negligible. Co released from leather is most probably related to the colour dye used. It was not possible to obtain detailed information about the dye composition from the tanneries; however, Co was included in the dye composition as reported in the material safety sheets. The significant difference between the two tanneries in terms of Co release points to different dye compositions among the tanneries and cross‐contamination of Co within one workplace.

With the extraction conditions of this study, which is optimized for the detection of Cr(VI), Co is not stable in solution and is expected to precipitate in the form of Co phosphate.^21^ Nevertheless, Co was clearly detected, especially for leathers from one of the tanneries. The detectable Co release corresponded to 1 to 5 mg/kg (ppm), which can be compared with the detected values of 1 to 190 ppm Co in five of 131 leather samples purchased in the United States, when they were analysed under conditions in which Co is expected to be soluble in solution.^22^ Our study is hence in agreement with that study,^22^ suggesting that leather, in some cases, contains and releases Co. It remains to be investigated whether leather is one of the possible causes of the known association between Co and Cr allergy in construction workers,^23^, ^24^ who are frequently exposed to leather (in work gloves and shoes) under conditions in which Cr(VI) is most likely to be released from leather.^12^, ^13^ A questionnaire study indicated an association between non‐occupational leather exposure and contact dermatitis caused by Co.^25^

There was no significant difference in total Cr or Cr(VI) release between the Nicaraguan and the European leathers. Total Cr release, including both trivalent and hexavalent Cr, was in all cases significantly higher (at least 10‐fold) than Cr(VI) release. The relative release of Cr(III) is even higher (at least 100‐fold greater release of Cr[III] than of Cr[VI]) under conditions of skin contact than in the phosphate buffer extraction test,^9^ as is evident from earlier studies on the European leathers.^6^, ^19^ It was also shown that the Cr(III) released under conditions of skin contact is able to elicit allergic contact dermatitis caused by Cr in sensitized individuals.^6^ It is therefore suggested that low release of Cr(VI) alone is not sufficient to protect Cr‐sensitized persons. There is also no association between Cr(VI) release in the REACH‐regulated extraction test and total Cr release. This suggests that the current restriction limit on the use of Cr(VI) in leather is not sufficient to protect Cr‐sensitized individuals.

## 5 CONCLUSION

This study analysed 29 differently coloured Cr‐tanned leather samples from two Nicaraguan tanneries, and compared their release of Cr, Cr(VI) and Co with that of three Cr‐tanned and one Cr‐free leather produced in Europe. There was no significant difference in total Cr or Cr(VI) release between European and Nicaraguan leathers. There was no association between Cr(VI) and total Cr release. The total amount of Cr released was 0.89 to 30.4 μg/cm^2^ and 12 to 635 mg/kg. Cr(III) was released in significantly higher amounts than Cr(VI), which was released at levels ranging from not detectable to 0.20 μg/cm^2^ and 6.0 mg/kg. Co was released primarily from the leathers of one tannery. Future investigations and regulations should focus on Cr(III) and Co as well as Cr(VI).

## CONFLICTS OF INTEREST

The authors have no conflicts of interest to report.
